# Clinicopathological and Molecular Analysis of 45 Cases of Pure Mucinous Breast Cancer

**DOI:** 10.3389/fonc.2020.558760

**Published:** 2021-03-01

**Authors:** Hyun Ee Yim, Jang-Hee Kim, Mi Sun Ahn, Yongsik Jung, Jin Roh, So Hyun Park, Tae-Gyu Kim, Jin-Hyuk Choi, Seok Yun Kang

**Affiliations:** ^1^ Department of Pathology, Ajou University School of Medicine, Suwon, South Korea; ^2^ Department of Hematology-Oncology, Ajou University School of Medicine, Suwon, South Korea; ^3^ Department of Surgery, Ajou University School of Medicine, Suwon, South Korea

**Keywords:** breast cancer, mucinous carcinoma, histological type, whole-exome sequencing, Immunohistochemistry

## Abstract

Pure mucinous breast carcinoma (PMBC) is characterized by clusters of tumor cells floating in abundant extracellular mucin and can be classified into paucicellular (Type A) and hypercellular (Type B) subtypes. However, the clinicopathological and genomic differences between these two subtypes have not been well characterized. We retrospectively investigated the clinicopathologic features of 45 cases of surgically removed PMBC (31 Type A and 14 Type B). We also performed whole-exome sequencing (WES) in eight cases of PMBC. We found that Type B PMBC occurs at an older age and shows more aggressive clinical behavior than Type A. WES analysis revealed that *HYDIN* was the most frequently mutated gene in both types of PMBC. Although Type B PMBC showed a tendency toward more frequent genetic alterations, there were no statistically significant differences between the two subtypes in single nucleotide variants or insertions or deletions of bases associated with moderate or high effects. Our results provide additional evidence that PMBCs are clinicopathologically and genetically heterogeneous and lack pathognomonic genetic alterations. Further, Type B PMBC is more frequently associated with lymph node metastasis than Type A.

## Introduction

Mucinous breast carcinoma (MBC) is a rare variant of breast cancer accounting for approximately 4% (range: 1 to 7%) of all breast carcinomas (1, 2). MBC most commonly occurs in elderly patients and is generally considered to have a favorable prognosis (1–3). MBC can be classified into pure and mixed types. Pure MBC (PMBC) is defined as MBC with more than 90% mucinous components, whereas mixed MBC includes more than 50% but less than 90% mucinous components, as well as other types of invasive ductal carcinoma (IDC) (2, 4). PMBC should be distinguished from mixed MBC because most PMBCs are detected at relatively early stages without lymph node (LN) metastasis, whereas mixed MBCs carry a prognosis similar to IDC (1, 3–5).

PMBCs are a heterogeneous group of tumors and can be further classified into two main subtypes by microscopic features of architecture and cytology: the “classical” variant, Type A (or paucicellular), with large quantities of extracellular mucin, and Type B (or hypercellular) tumors, which contain less mucin and often show histologic features significantly overlapping with those of neuroendocrine carcinomas (6, 7). PMBC is typically estrogen receptor (ER)-positive and human epidermal growth factor receptor 2 (HER2)-negative. However, PMBC is molecularly different from ER-positive/HER2-negative IDC-not otherwise specified (NOS), which frequently harbors gains of 1q and 16p and losses of 16q and 22q (4, 8, 9).

While recent advances in DNA sequencing technologies have provided substantial insights into the mutated cancer genes and mutational processes operative in breast cancer (10), data on pathognomonic genetic alterations of PMBC are still limited (8). It has been reported that Type A PMBC could be a subgroup distinctive from Type B, which displays gene expression patterns similar to neuroendocrine carcinoma of the breast (9). However, other studies revealed no significant difference in gene expression between the two subtypes (2, 4, 8).

In the present study, we investigated the clinicopathologic features of 45 cases of surgically removed PMBC and performed whole-exome sequencing (WES) in eight cases of PMBC in an attempt to characterize PMBC and improve patient stratification.

## Materials and Methods

### Case Selection

We retrospectively reviewed the pathology records of breast cancer patients that were submitted to the pathology department of the Ajou University Hospital from April 2011 to April 2017. We included surgical specimens from partial, modified radical, or total mastectomy and excluded biopsy specimens. Among 2006 cases of breast cancer, we included MBC and excluded mixed MBCs and IDCs. Finally, we found 45 cases of PMBC.

### Immunohistochemistry

Immunohistochemistry was conducted on representative sections (4 µm thick) of formalin-fixed, paraffin-embedded (FFPE) tissues using a BenchMark XT automated immunohistochemistry stainer (Ventana Medical Systems, Inc., Tucson, AZ, USA) as previously described (11). Briefly, the primary antibodies used were as follows: CONFIRM anti-ER (SP1), prediluted (Catalog No: 790-4347, Ventana Medical Systems, Inc.); CONFIRM anti-progesterone receptor (1E2), prediluted (Catalog No: 790-2223, Ventana Medical Systems, Inc.); VENTANA anti-HER2/neu (4B5), prediluted (Catalog No: 790-4493, Ventana Medical Systems, Inc.); anti-synaptophysin, 1:100 (Catalog No: 336R-95, Cell Marque Co., Rocklin, CA, USA); anti-Human Ki-67 (clone MIB-1), 1:100 (Catalog No: M7240, DAKO, Glostrup, Denmark). Detection was performed using the Ventana Optiview DAB Kit (Ventana Medical Systems, Inc.). Slides were incubated with hematoxylin and a bluing reagent (4 min each). We removed the slides from the immunostainer and washed them in water containing a drop of dishwashing detergent. Finally, the slides were mounted. The immuno-expression was evaluated according to the American Joint Committee on Cancer (AJCC) cancer staging manual (12) by a single experienced pathologist (JH Kim) without prior knowledge of the clinicopathological data. For ER and progesterone receptor (PR), any staining of 1% of cells or more was considered positive. The HER2 immunostaining was performed using a pattern of membrane staining and graded as 0, 1+, 2+, and 3+. Grade 0 and grade 1+ were considered negative and grade 3+ was considered positive. Grade 2+ classified as equivocal. For Ki-67, nuclear immunostaining was considered as positive and the Ki-67 index was measured as the percentage of positively stained cells among the total number of malignant cells. The analysis was performed at three randomly selected, high-power (×40 objective) fields. For analysis, at least 500 tumor cells were counted.

### Whole-Exome Sequencing Analysis

Among 45 cases of PMBC, eight (4 Type A and 4 Type B) representative cases were selected for WES. Eight to 10 representative 10 μm thick FFPE tissue sections of the tumors were obtained from each specimens. Microdissection of tumors was performed by an experienced pathologist with a sterile needle under a stereomicroscope (Olympus SZ61, Tokyo, Japan). DNA extraction from micro-dissected tumor samples was performed separately using the GeneRead™ DNA FFPE Kit (Qiagen, Hilden, Germany), according to the manufacturer’s guidelines. The quality and quantity of purified DNA were analyzed using fluorometry (Qubit, Invitrogen, Carlsbad, CA, USA) and gel electrophoresis.

We performed WES analysis as previously described (13). Briefly, we performed a fragmentation of 500 ng of genomic DNA from each sample by acoustic shearing on a Covaris S2 instrument. Ligations of fragments of 150–300 bp to Illumina adapters and PCR-amplification were processed. We concentrated the samples to 300 ng in 3.4 μl DW using a Speedvac (Thermo Fisher Scientific, Waltham, MA, USA) and conducted hybridization to RNA probes using SureSelect XT Human All Exon V5 Capture library (Agilent Technologies, Inc., Santa Clara, CA, USA) for 16–24 h at 65°C. After hybridization, the probe/target hybrids were captured by magnetic beads (Dynabeads MyOne Streptavidin T1, Thermo Fisher Scientific) and buffers. We performed PCR-amplification of the selected regions using Illumina PCR primers. Quantifications of libraries were processed using the Agilent 2100 Bioanalyzer (Agilent Technologies, Inc.) and KAPA Library Quantification Kit (KK4824, Kapa Biosystems, Inc., Wilmington, MA, USA). After the quantifications of libraries, we applied the purified libraries to an Illumina flow cell for cluster generation and sequenced using 100 bp paired-end reads on an Illumina Hiseq2500 sequencer following the manufacturer’s protocols. Image analysis was conducted using the HiSeq Control Software version 1.8.4. (Illumina Inc., San Diego, CA, USA).

### Sequencing Quality Control

The quality of the reads was checked using FastQC (v.0.11.7) (14), which provides information on basic quality for the sequence quality score, GC content, N content, length distribution, and duplication level. After checking the read quality, low-quality bases (below Q20) were trimmed using Trimmomatic (v.0.36) (15).

### Sequence Alignment

High-quality reads were aligned to the human reference genome hg19 using Burrows-Wheeler Aligner (v.0.7.17) (16) with a minimum seed length of 45. After aligning the reads to the reference genome, duplicated reads were further removed using Genome Analysis Toolkit (v.4.0.11) (17).

### Variant Call and Annotation

Germline and somatic variant calling were performed with Mutect2 using GATK best practices pipeline recommended commands and arguments. SNPs and INDELs were called from every alignment file using HaplotypeCaller with GVCF mode, which covers variable sites and the reference genotype, and then all g.vcf files were merged using CombineVCF. The types of variants were annotated to the GRCh38 human reference genome (ENSEMBL) using snpEff (ver. 4.3t) (18) with respect to predicting damage or clinical effect of the variants using dbNSFP, COSMIC(v.85) (19), and ClinVar (20). Allele frequencies were referred from 1000 Genomes (21), ESP6500 [W. NHLBI GO Exome Sequencing Project (ESP) Seattle] exome variant server, which is available online at http://evs.gs.washington.edu/EVS/(2013) (Accessed: 03/18/2013), and the ExAC database (22). To eliminate false positives, the column value in the vcf file was filtered as “PASS” and only the overlapped variant was selected from at least two samples. Additionally, Mutations that are common in gnomaAD (23) mutants with a minor allele frequency greater than 0.05 were removed to eliminate any known normal mutations.

### CNV Analysis

CNVs were analyzed using CNVkit (24). The mean value of total coverage was applied for a baseline for copy number calling on the tumor cohort. Copy numbers were calculated according to the depth of coverage in each region with the correction of GC bias and regional bias. CNVKit reports copy number as a log2 ratio change. CNVs were reported if the absolute copy number was above 6 [log2 (6/2) = 1.58] or below 1 [log2 (1/2) = − 1].

### Tumor Mutational Burden

TMB was defined as the number of nonsynonymous coding mutations per megabase (the total number of nonsynonymous mutations in a coding region divided by the length of the total genomic target region captured with the exome assay × 1,000,000). For TMB analysis, false positive germline variants were filtered out using the ExAC database (22) after germline and somatic variant calling with Mutect2 using GATK best practices pipeline recommended commands and arguments. In addition, the variants included in COSMIC(v.85) (19) were removed to avoid bias toward genes with functional mutations in cancer.

### Analysis of Somatic Genetic Alterations Between Type A and Type B Pure Mucinous Breast Carcinoma

For somatic variant calling, all variants were annotated by SnpEff (v.4.3t) in the context of predicting damage or clinical effect of the variants using dbNSFP (25), COSMIC(v.85) (19), and ClinVar (20). Allele frequencies were referred from 1000 Genomes (21), ESP6500 [W. NHLBI GO Exome Sequencing Project (ESP) Seattle] exome variant server, which is available online at http://evs.gs.washington.edu/EVS/(2013) (Accessed: 03/18/2013), and the ExAC database (22). To eliminate false positives, the column value in the vcf file was filtered as “PASS” and only the overlapped variant was selected from at least two samples. Additionally, Mutations that are common in gnomaAD (23) mutants with a minor allele frequency greater than 0.05 were removed to eliminate any known normal mutations. We compared the frequencies of both the reference and an alternative allele for variable loci in both types of PMBC. In addition, only the variants that are associated with cancer by oncotator (https://github.com/broadinstitute/oncotator) were selected. Differences in somatic mutations between Type A and B PMBC were visualized as an oncoplot.

### Statistical Analysis

Student t-test and Fisher’s exact test were performed to compare differences in clinicopathological parameters and frequencies in both reference and alternative alleles for variable loci. Statistical significance was defined as *p <*0.05. Statistical analyses were performed using R v3.1.2.

## Results

### Clinicopathologic Characteristics of Pure Mucinous Breast Carcinoma

**Figure 1:**
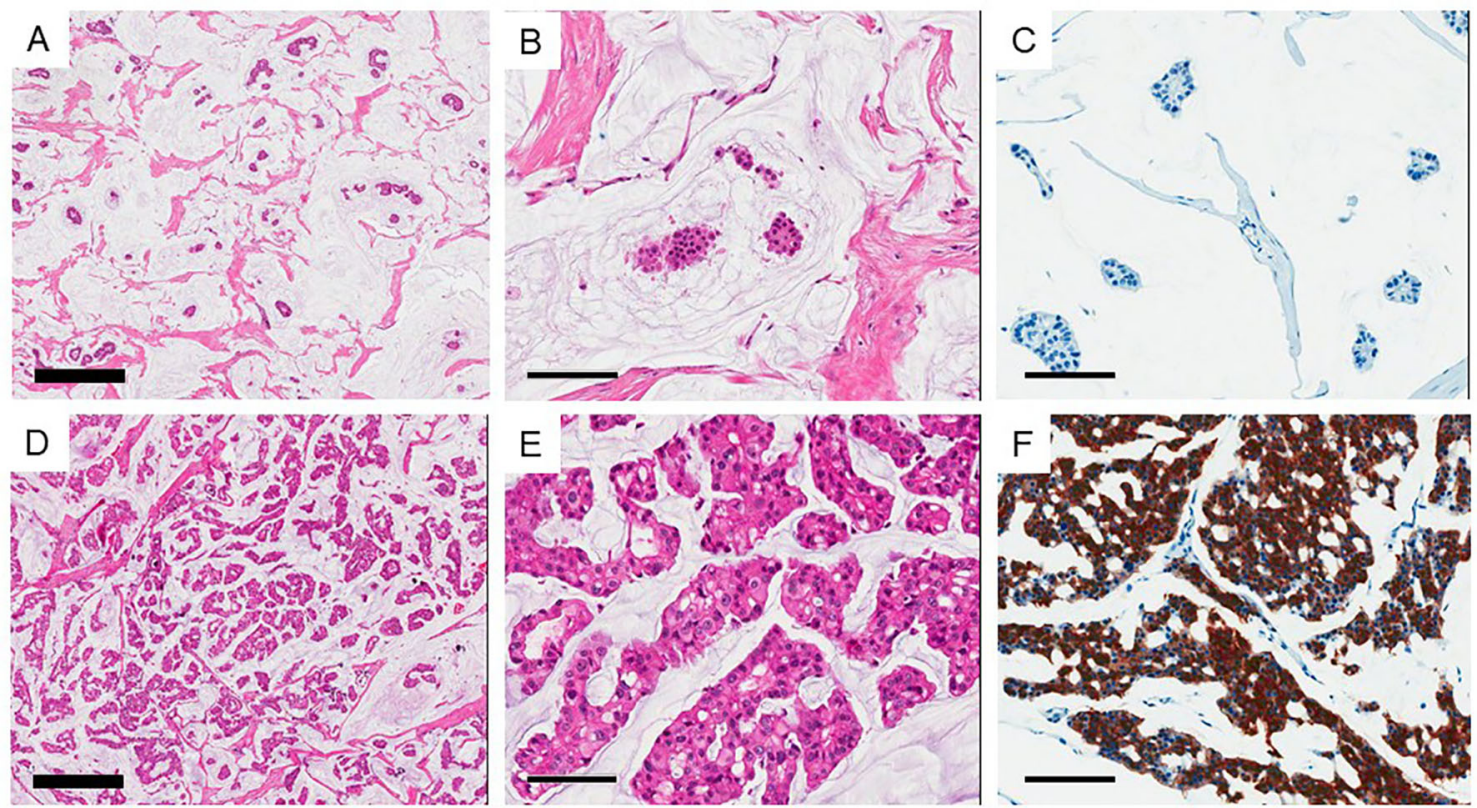
Two subtypes of mucinous carcinoma. **(A–C)** Type A (Paucicellular) variant and **(D–F)** Type B (hypercellular) variant. **(B, E)** high power view of each variant. **(C, F)** Show immunohistochemical staining of synaptophysin. Thick bar 500 μm and thin bar 100 μm.

Of the 45 patients with PMBC, 31 had classic paucicelluar (Type A) and 14 had hypercellular (Type B) PMBC (**Figure 1**, **Table 1**, and **Supplementary Table 1**). All the patients were women, and the median age was 47 years (range: 29–85 years). The median age of patients with Type B PMBC (51 years) was older than that of patients with Type A PMBC (43 years). The mean tumor size (greatest dimension) of Type B was slightly larger than that of Type A (A: 1.9 ± 1.0 cm vs B: 2.3 ± 1.5 cm). Among the 45 patients, ductal carcinoma *in situ* was presented adjacent to PMBC in 17 cases (37.8%). Among the 14 Type B cases, 3 (21.4%) revealed neuroendocrine differentiation, whereas only 1 Type A case (3.2%) was positive for neuroendocrine markers (**Figure 1**). The mean Ki-67 index was slightly higher in Type B than Type A. However, it was not significantly different (*p* = 0.406). All cases were positive for hormone receptors and negative for HER2. LN metastasis occurred in three cases (6.7%), all of which were Type B PMBC. Type B PMBCs showed a tendency toward more advanced stage disease than Type A. However, among these factors, only LN metastasis was significantly more frequent in Type B than in Type A PMBC (*p* = 0.026) (**Table 1**). All patients were alive at the time of analysis, and one patient with Type B underwent reoperation due to local recurrence.

**Table 1:** Clinicopathologic characteristics of patients in two pathologic subtypes.

	Paucicellular (Type A)	Hypercellular (Type B)	*p* value^*^
Patient (*n*)	31	14	
Median age (range, years)	43 (29–73)	51 (40–85)	0.055^†^
Menopausal status (*n*)			0.286
Premenopausal	24	8	
Postmenopausal	7	6	
Tumor Characteristics			
Mean size ( ± SD, cm)	1.94 ( ± 0.98)	2.36 ( ± 1.49)	0.260^†^
Multiple foci (*n*)	3	0	0.541
Presence of DCIS (*n*)	10	7	0.326
ER positive (*n*)	31	14	–
PR positive (*n*)	31	14	–
HER2 positive (*n*)	0	0	–
Synaptophysin positive (*n*)	1	3	0.082
Mean Ki-67 index ( ± SD, %)	11.54 ( ± 8.46)	13.89 ( ± 9.15)	0.406^†^
Histologic grade (*n*)			0.111
Grade I	15	3	
Grade II	15	9	
Grade III	1	2	
Nuclear grade (*n*)			0.665
Grade I	3	2	
Grade II	22	11	
Grade III	6	1	
LN metastasis (*n*)			0.026
Absence	31	11	
Presence	0	3	
AJCC Stage (8ed) (*n*)			0.053
IA	20	5	
IIA	10	6	
IIB	1	3	

n, Number of cases; SD, Standard deviation; DCIS, Ductal carcinoma in situ; ER, Estrogen receptor; PR, Progesterone receptor; HER2, Human epidermal growth factor receptor 2; LN, Lymph node; AJCC, American Joint Committee on Cancer.

^*^p values of each parameters were analyzed by Fisher’s exact test except for age, tumor size and Ki-67 index.

^†^Analysis was performed by Student t test.

### Whole-Exome Sequencing Analysis of Eight Cases of Pure Mucinous Breast Carcinoma

**Figure 2:**
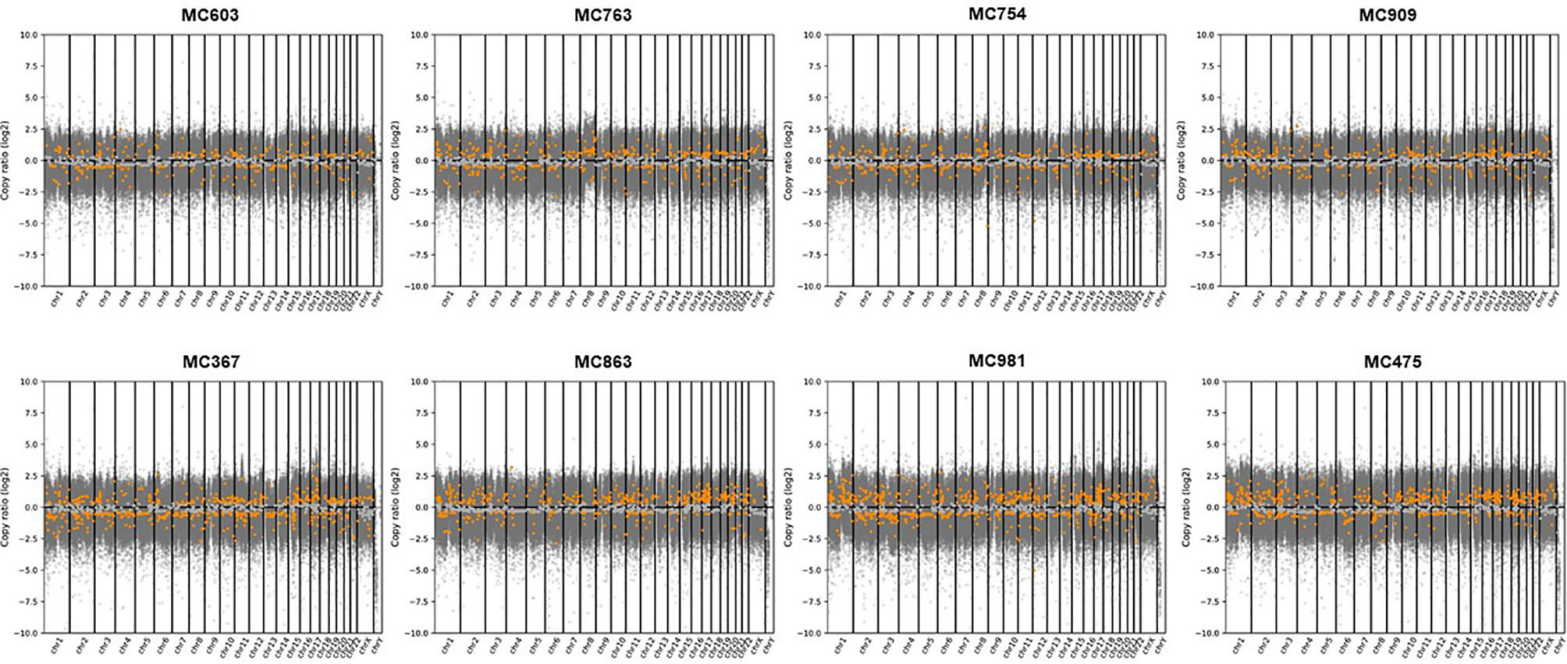
Copy number alterations analyzed by CNVkit in eight representative cases of PMBCs subjected to WES. Copy numbers of genes in each PMBC were plotted segmented log2 ratios (y-axis) according to their genomic positions (x-axis). Gray spots indicate mean copy number and orange spots mean gain or loss of genes.

By WES analysis, we found a total of 2,842,224 single nucleotide variants (SNVs) and 384,759 insertions or deletions of bases (INDELs) in eight representative cases of PMBC (four Type A and four Type B). Most of these genetic alterations were located in introns and intergenic regions. In the coding region, 51.5% of SNVs were synonymous, whereas 48.0 and 0.5% were missense and nonsense mutations, respectively. Among these mutations, 2,363 SNVs (0.045%) and 1,332 INDELs (0.184%) were classified as having a high (disruptive) impact, causing protein truncation, loss of function, or triggering nonsense-mediated decay (**Supplementary Figure 1**). At the copy number level, we observed a putative gain of chromosome 8 in one out of eight cases (MC763). However, we could not find any other high-level copy number changes such as concurrent 1q whole-arm gains or 16q whole-arm losses, which are hallmark genetic alterations of ER-positive/HER2-negative breast cancers (**Figure 2**, **Supplementary Table 2**–**9**). The mean TMB was 4.7/Mbps (range 3.5–5.0/Mbps).

### Comparison of Genetic Alterations in Type A and Type B Pure Mucinous Breast Carcinoma

**Figure 3:**
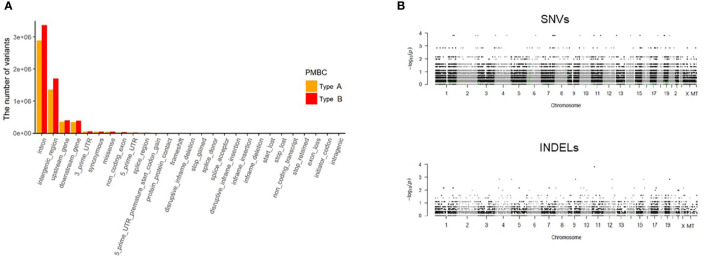
Analysis of WES data according to classic paucicellular (Type A) and hypercellular (Type B) PMBC. **(A)** Regional differences of variants in Type A and B PMBC **(B)** Manhattan plot of single nucleotide variants (SNVs) and of insertions or deletions (INDELs). The y axis represents log-scaled *p* values.

**Figure 4:**
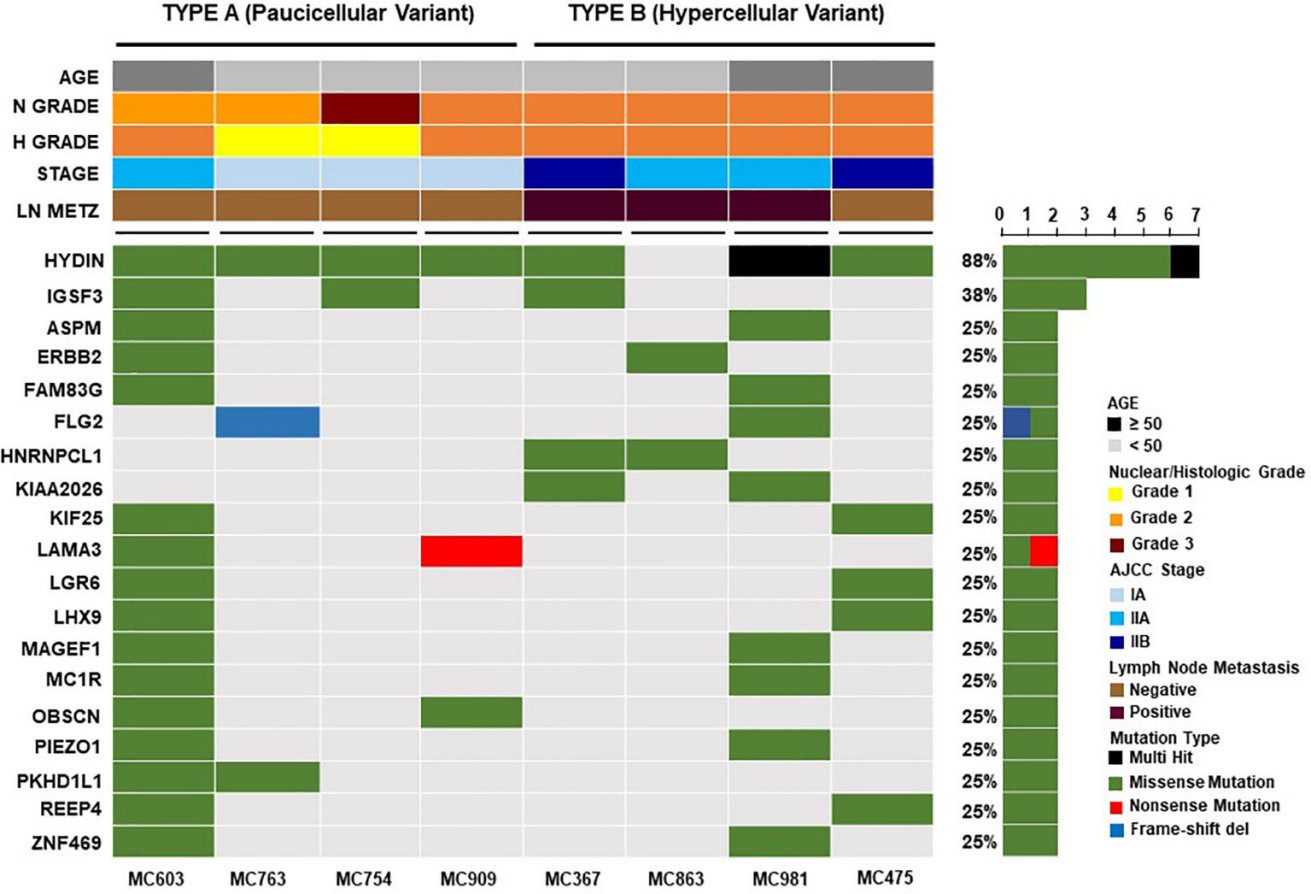
Recurrent somatic genetic alterations in mucinous breast cancers. Each column represents a sample and each row a different gene. The top barplot depicts clinicopathologic characteristics for each patient, while the right barplot shows the frequency of mutations in each gene. Genetic alterations are colored according to the variant type as indicated in the legend. Genes annotated as “Multi-Hit” have more than one mutation in the same sample.

We analyzed differences in frequencies of both reference and alternative alleles for variable loci between Type A and Type B PMBC. Although Type B PMBC showed a tendency toward more frequent genetic alterations compared to Type A, there were no statistically significant differences in SNVs or INDELs associated with moderate or high effects between the two subtypes (**Figure 3**). We further investigated recurrent somatic mutations associated with cancer. We only selected somatic mutations identified in at least two patients and found a total of 19 genes. Among them, *HYDIN* (88%) was the most frequently mutated gene (**Figure 4**), followed by *IGSF3* (38%). *ERBB2* mutations, not amplification, were identified in two cases of PMBC (one was Type A and the other was Type B). *LAMA3*, *OBSCN*, and *PKHD1L1* were identified only in Type A PMBC whereas *HNRNPCL1* and *KIAA2026* were found only in Type B.

## Discussion

In the present study, we investigated the clinicopathologic features of 45 cases of surgically removed PMBC and found that the clinicopathological parameters of both types of PMBC were consistent with the results of previous studies, except the age of the patients. The median age of the patients was younger than those reported in previous studies (1–3, 5, 26). The difference can be associated with geographical and ethic variations. It has been reported that the mean age of patients at diagnosis of breast cancer in Asian populations is lower than that in Western populations (27–29). A large series of mucinous cancer study in Korea also revealed that the mean age of the patients was younger than those of previous reports (30). LN metastasis in PMBC has been reported in 2 to 14% of cases and is suggested to be the most significant predictor of disease-free survival (1, 5, 31, 32). In the present study, there were only three cases (6.67%) of PMBC with LN metastasis and one case (2.2%) of recurrence. Interestingly, all of these cases were Type B (hypercellular). The risk of LN metastasis in PMBC could increase in association with larger tumor size, a smaller proportion of mucinous component, and p53 expression (2, 3). However, the association between LN metastasis and PMBC subtype has not been clearly investigated. Lei et al. (3) reported no significant differences in the clinicopathological characteristics between the two types of PMBC. However, other studies (7, 33) and our results showed that Type B PMBC was associated with more frequent LN metastasis than Type A, providing evidence that the subtype may also be a risk factor of LN metastasis in PMBC. However, due to the limited number of cases in this study, more investigations with larger cohorts should be performed to confirm the results. Compared to Type A PMBC, Type B displays distinctive transcriptomic profiles strikingly similar to those of neuroendocrine carcinomas, suggesting that the two subtypes of MBC may arise from different cells of origin (4, 9). It has been reported that Type B PMBC harbors gains of 2q37 and 11q13 significantly more frequently than Type A (4). However, no statistically significant differences in amplification or the mutational repertoire between Type A and Type B PMBC have been identified (4, 8). In the present study, we found that Type B PMBC showed not only more frequent neuroendocrine differentiation but also higher genetic alterations than Type A. However, we failed to find any significant differences in SNVs or INDELs between the two subtypes of PMBC or any specific molecular changes for Type B PMBC.

PMBC, unlike other types of ER-positive/HER2-negative breast cancer, usually does not show concurrent 1q whole-arm gains and 16q whole-arm losses (4, 8). In the present study, all 45 cases of PMBC were ER-positive and HER2-negative. By CNV analysis, we observed a putative gain of chromosome 8 in one out of eight cases. However, as in previous studies (4, 8), we could not observe other changes such as concurrent 1q whole-arm gains and 16q whole-arm losses, a hallmark genetic feature of invasive ductal carcinomas of no special type. Mucinous cancers of other organs are commonly associated with microsatellite instability (MSI) (34) and, as a result, have a high mutation burden (35–38). However, MBCs are rarely associated with MSI (34) and have a low level of genetic instability, rare recurrent amplification, and often display a relatively simple genomic pattern (4, 8, 10, 39). In the present study, we also observed a relatively low mutation burden in PMBC, supporting the findings of previous studies.

Recent studies with high-throughput technologies (8, 40) revealed that PMBC had a low frequency of *PIK3CA* mutations. Pareja et al. (8) suggested that *GATA3*, *KMT2C*, and *MAP3K1* were the most frequently mutated genes in PMBCs. However, Nguyen et al. (40) reported that deletion of *RB1*, *BRCA2, EGFR, CDH1, TP53, MAP2K4, and PGR* were most frequently identified. In the present study, we also observed a low frequency of *PIK3CA* mutations. However, we found *HYDIN* (88%) was the most frequently mutated somatic gene in PMBC. *HYDIN* (axonemal central pair apparatus protein) is known to encode a protein involving in cilia motility and mutations in this gene have been suggested as a cause of autosomal recessive primary ciliary dyskinesia-5 (41). Mutations of *HYDIN* were also identified in breast cancers (42, 43). In addition, a recent study suggested that mutations in the *HYDIN* gene may be associated with the tumorigenesis of neuroendocrine tumors (44). However, Pongor et al. (45) suggested that, despite the high mutation rate, the *HYDIN* gene was not included in the list of putative driver genes due to a tendency of high false discovery rate in mutation calling. Therefore, further study should be performed to elucidate the biologic role of *HYDIN* in the carcinogenesis of pure mucinous breast cancer.

In the present study, we found *ERBB2* mutations (NM_004448.3:c.3436C>T, NM_004448.3:c.428G>A) in two out of eight cases of PMBC. *ERBB2* mutations are uncommon (2.2%) and mostly identified in ductal carcinoma (46). Moreover, *ERBB2* mutations identified in the present study have not been reported in the breast cancer previously. *ERBB2* mutation is not mutually exclusive of ERBB2 amplification and could be accompanied with *ERBB2* amplification (46, 47). However, in the present study, we could not find any coexistence of mutation and amplification of *ERBB2* in PMBC. A recent study suggested that *ERBB2* activating mutations could be responsive to HER2 tyrosine kinase inhibitors (47). Therefore, the examination of *ERBB2* mutations could provide additional treatment options in case of aggressive PMBC without *ERBB2* amplification.

There are several limitations to this study. First, this is a retrospective study conducted in a single institution. Since the study included only those with PMBC over a 6-year period, the number of patients was very small, and the treatment procedure was not uniform. Second, research using medical records reduces data accuracy and lacks patient follow-up. Finally, it is difficult to generalize the results because we performed WES in only a few patients. Despite these limitations, it is meaningful that this study identified differences in clinicopathological characteristics in patients with the two types of PMBC.

## Conclusion

Our results provide additional evidence that PMBCs are clinicopathologically and genetically heterogeneous and lack pathognomonic genetic alterations. Type B PMBC is more frequently associated with LN metastasis than Type A. However, due to the limited number of cases in this study, more investigations with larger cohorts should be performed to confirm the results.

## Data Availability Statement

The datasets presented in this article are not readily available because patients have not explicitly consented for the public release of their genomic data. Requests to access the datasets should be directed to SK, kangsy01@ajou.ac.kr.

## Ethics Statement

The studies involving human participants were reviewed and approved by the Ajou Institutional Review Board Committee (Ajou IRB, Approval No. AJIRB-BMR-KSP-17-106). Written informed consent for participation was not required for this study in accordance with the national legislation and the institutional requirements.

## Author Contributions

SYK and J-HK conceptualized the study concept and design. MSA, J-HC, and YSJ provided the patient samples and clinical data analysis. J-HK and HY were in charge of the immunohistochemistry and interpretation. JR, SHP, and T-GK performed the molecular experiments and analysis. SYK and J-HK drafted the manuscript. All authors contributed to the article and approved the submitted version.

## Funding

This work was supported by the faculty research fund (2018 Clinical-Basic Intermediary Cooperation Research) of Ajou University School of Medicine to SYK, National Research Foundation of Korea to J-HK (NRF-2016R1D1A1B02010452), and CJ Healthcare Corporation, Korea. The funders did not have any role in the design and conduct of the study, the analysis and interpretation of the data, and preparation of the manuscript.

## Conflict of Interest

The authors declare that the research was conducted in the absence of any commercial or financial relationships that could be construed as a potential conflict of interest.
