# Supplementary material for: Clinicopathological and Molecular Analysis of 45 Cases of Pure Mucinous Breast Cancer
Source: Front Oncol. 2021 Mar 1;10:558760. doi: 10.3389/fonc.2020.558760 (PMC7956951; doi:10.3389/fonc.2020.558760)
Supplement: Supplementary file 1 [file Presentation_1.pptx]

## Slide 1
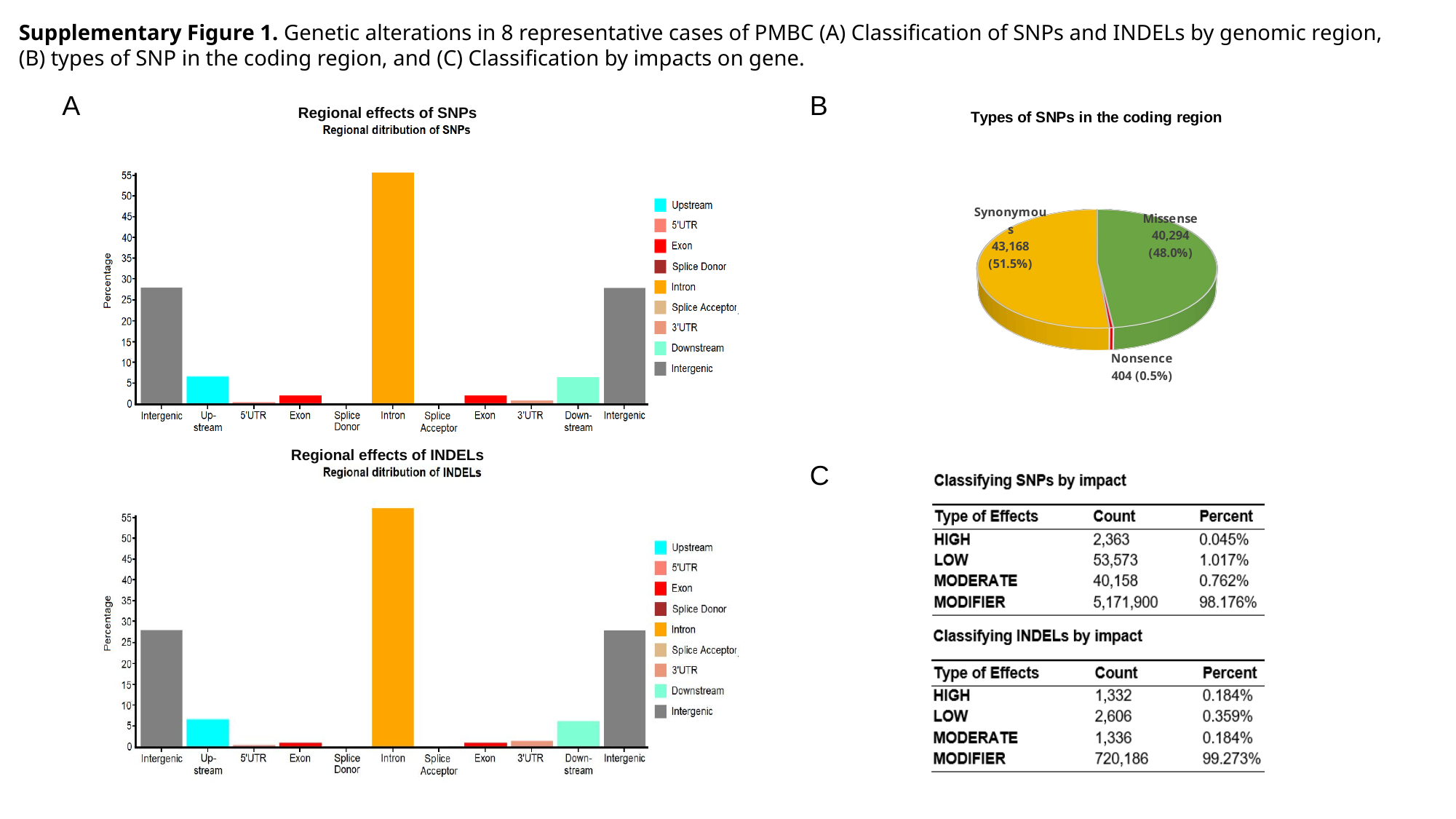

Supplementary Figure 1. Genetic alterations in 8 representative cases of PMBC (A) Classification of SNPs and INDELs by genomic region,
(B) types of SNP in the coding region, and (C) Classification by impacts on gene.
A
B
[unsupported chart]
Regional effects of SNPs
Regional effects of INDELs
C
